# Clinical efficacy and bleeding outcomes of tissue plasminogen activator and dornase alfa in pleural space infection with once daily concurrent administration: a retrospective cohort study

**DOI:** 10.1186/s13104-020-05210-2

**Published:** 2020-08-03

**Authors:** Chuan Jiang, Meng Xie, Kelly Cervellione, Craig Thurm

**Affiliations:** 1 Department of Medicine-Division of Pulmonary Medicine, Jamaica Hospital Medical Center, Jamaica, NY 11418 USA; 2 Department of Clinical Research, Jamaica Hospital Medical Center, Jamaica, NY 11418 USA

**Keywords:** Parapneumonic effusion, Empyema, Fibrinolytic therapy, Pleural disease

## Abstract

**Objective:**

The use of intrapleural tissue plasminogen activator (tPA) and dornase alfa (DNase) is common in the management of pleural space infection. We review our experience with the efficacy and safety of this therapy. We performed a single center, retrospective study of consecutive patients with complicated parapneumonic effusion or empyema who received tPA/DNase therapy. Treatment success was defined as radiographic and clinical improvement in pleural space infection that precluded the need for surgical intervention, and the absence of mortality related to pleural infection.

**Results:**

Fifty-six patients received concurrent once daily tPA/DNase therapy (median 3 days) from July 2014 to July 2019. Fifty-two patients (92.9%) had treatment success. Median duration of chest tube therapy was 10 days and length of stay was 15 days. Significant pleural bleeding requiring transfusion therapy occurred in five patients (8.9%). Of these, three patients (5.4%) required operative intervention. Concurrent once daily administration of tPA/DNase in patients with pleural infection yielded comparable rates of treatment success as compared to twice daily concurrent or sequential administration. However, adverse events highlight potential safety concerns with using once daily concurrent administration of tPA/DNAse.

## Introduction

Empyema and complicated parapneumonic effusions (CPE) are estimated to cost the United States healthcare system at least 500 million dollars a year [1] and the former is associated with a mortality of at least 15% [2]. Fibrinolytic therapy for intrapleural infections has been available since 1949, when it was initially described by Tillet and Sherry, as a therapy to facilitate improved drainage of infected pleural effusions [3].

The use of intrapleural alteplase (tPA) and dornase alfa (DNase) in the management of complicated parapneumonic effusions and empyema was demonstrated to improve chest tube drainage, reduce the requirement for thoracic surgical intervention, and reduce hospital length of stay as compared to placebo and alteplase use alone [4].

Intrapleural hemorrhage requiring blood transfusion was observed in 3.8% of the patients receiving combination therapy [4]. In a “real world” study, pleural bleeding requiring blood transfusion also occurred at a similar 1.8% rate [5]. At our institution, we utilized a once daily concurrent administration technique and experienced good clinical efficacy. However, we observed a higher rate of significant pleural bleeding as compared to the above studies.

## Main text

### Methods

We reviewed the charts of all patients admitted to our hospital with complicated parapneumonic effusion or empyema from January 1, 2014 to July 1, 2019. Patients were identified using ICD codes and pharmacy records. Patients were included if there was: (1) a complicated parapneumonic effusion defined by a non-purulent effusion in a patient with pleural fluid pH less than or equal to 7.2, pleural fluid glucose less than 60 mg/dL, pleural fluid lactate dehydrogenase (LDH) greater than 1000 IU/L, and/or radiographic evidence of loculations; or (2) empyema defined by purulent pleural fluid or microbiological identification within the fluid [6].

The dose of DNase (Pulmozyme; Genetech, South San Francisco, CA) was 5 mg in 30 mL of sterile water and the dose of tPA (Activase; Genetech, South San Francisco, CA) was 10 mg in 10 mL of 0.9% NaCl. Concurrent administration (i.e. injection of tPA followed by DNase one after the other) of these agents was followed by a 10 mL saline flush. The chest tube was clamped for 60 min before it was opened to a gravity bag. Routine therapy was given once daily. The total number of days of administration was at the discretion of the treating physician.

In contrast, the sequential administration method refers to: (1) the administration of tPA followed by a 10- to 25-mL flush of saline followed by a 60 min clamp, (2) an unclamp followed by a 60 min open drainage period, (3) the administration of DNase followed by a 10- to 25-mL flush of saline followed by another 60 min clamp, and (4) an unclamp to open drainage.

Electronic medical records were reviewed for patient demographics, clinical characteristics, treatment, outcomes, and safety. The primary efficacy outcome was treatment success, which was defined as clinical and radiologic improvement without the need for surgical intervention or mortality. Clinical and radiographic improvement were assessed by documentation by the treating physician. Radiographic improvement was defined as significant improvement of the opacified hemithorax on chest x-ray. Other clinical outcome measures included need for operative intervention for treatment failure, need for operative intervention for pleural bleeding complications, mortality related to pleural infection or bleeding complications, cumulative volume of pleural fluid drainage, length of chest tube therapy, and length of hospital stay.

Incidence of major bleeding was defined according to the International Society of Thrombosis and Haemostasis [7] as (1) Fatal bleeding, and/or (2) Symptomatic bleeding in a critical organ such as intracranial, intraspinal, intraocular, retroperitoneal, intraarticular, intrathoracic, pericardial, or intramuscular with compartment syndrome, and/or (3) Bleeding causing a fall of hemoglobin of 2 g/dL or more leading to a transfusion of two or more units of packed red blood cells (pRBC).

Further, we defined “pleural bleeding” as a change in chest tube drainage to a bloody appearance accompanied by signs and symptoms of bleeding (i.e. worsening respiratory symptoms, incident pleuritic chest pain, worsening radiographic appearance, and/or decrease in serum hemoglobin values). Evidence of pleural bleeding found during surgery was also considered “pleural bleeding.”

Statistical analysis was performed using Microsoft Excel. Descriptive statistics for continuous data are expressed as mean (standard deviation [SD]) if normally distributed and as median (interquartile range [IQR]) if non-normally distributed; for categorical data statistics are expressed as N (%). The study was approved by the Jamaica Hospital Medical Center Institutional Review Board (IRB# 1527223).

### Results

#### Patients

Our study included 56 patients with CPE or empyema who received concurrent tPA/DNase therapy. All patients meeting inclusion criteria during the defined time period were included. Complete data was available for all patients. The baseline demographics and pre-existing medical conditions of these patients are outlined in Table 1. The mean age was 64.8 (± 16.2) years with 64.2% being male. Twenty-one patients (37.5%) were on an antiplatelet agent or anticoagulant. Only five (8.9%) were on full dose anticoagulation for a pre-existing indication.

**Table 1:** Patient demographics and comorbidities

Mean age (SD), year	64.8 (16.2)
Male, n (%)	36 (64.2)
Racial background, n (%)	
Caucasian	21 (37.5)
Black	11 (19.6)
Hispanic	10 (17.9)
Asian	12 (21.4)
Other/mixed	2 (3.6)
Comorbidities, n (%)	
Hypertension	39 (69.6)
Diabetes mellitus	26 (46.4)
Coronary artery disease	13 (23.2)
Chronic kidney disease	12 (21.4)
Gastroesophageal reflux disease/peptic ulcer disease	10 (17.9)
Congestive heart failure	10 (17.9)
Cerebrovascular accident	9 (16.1)
End stage renal disease on hemodialysis	8 (14.3)
Atrial fibrillation	7 (12.5)
Asthma	4 (7.1)
Substance related condition	4 (7.1)
Chronic obstructive pulmonary disease	3 (5.4)
Valvular heart disease	2 (3.6)
Hepatitis B or C	1 (1.8)
Common variable immunodeficiency	1 (1.8)
Bronchiectasis	0
Active malignancy	0
Anticoagulation or antiplatelet medications prior to admission, n (%)	
Aspirin only	12 (21.4)
Aspirin and clopidogrel	4 (7.1)
Rivaroxaban	2 (3.6)
Apixaban	1 (1.8)
Enoxaparin	1 (1.8)
Warfarin	1 (1.8)

Clinical, radiographic, microbiological, and treatment data are shown in Table 2. Evidence of loculations on CT chest imaging were noted in 78.6% of the patients. Purulence of pleural fluid was noted in 30.4% of patients and 46.4% of the sampled effusions yielded a positive bacterial culture. The predominant microorganisms isolated were *Streptococcus milleri* (n = 8) and *Staphylococcus aureus* (n = 9; methicillin sensitive, n = 5; methicillin resistant n = 4). The majority of patients received three or fewer doses of tPA/DNase (92%). The reasons for using fewer than three doses varied on a case by case basis, including rapid resolution of clinical and radiographic features (n = 6), patient refusal due to intractable chest pain (n = 3), and change in hemodynamics (n = 2).

**Table 2:** Clinical data

Presence of loculations on CT scan, n (%)	44 (78.6)
Number of chest tubes placed	
1 chest tube placed, n (%)	53 (94.6)
2 chest tubes placed, n (%)	3 (5.4)
Size of chest tubes placed	
32 French, n (%)	3 (5.1)
12 French, n (%)	7 (12.5)
10 French, n (%)	39 (69.6)
8 French, n (%)	10 (17.8)
Purulent pleural fluid, n (%)	17 (30.4)
Positive pleural fluid culture, n (%)	26 (46.4)
Organisms found on pleural fluid culture	
*Streptococcus milleri*	8 (30.8)
*Klebsiella pneumoniae*	5 (19.2)
*Staphylococcus aureus*, methicillin sensitive	5 (19.2)
*Staphylococcus aureus*, methicillin resistant	4 (15.4)
*Escherichia coli*	3 (11.5)
*Streptococcus pneumoniae*	2 (7.7)
*Enterobacter aerogenes*	1 (3.8)
*Enterococcus faecium*	1 (3.8)
*Gemella* sp	1 (3.8)
Median pleural fluid pH (IQR)	7.08 (6.87–7.18)
Median pleural fluid lactate dehydrogenase (IQR), IU/L	4456.5 (1975.8–12,762.5)
Median peripheral leukocyte count (IQR), × 10^9^/L	15.9 (12.6–20.5)
Median ESR (IQR), mm/h	83.5 (57.5–101.3)
Median CRP (IQR), mg/L	8.25 (4.8–16.6)
Antibiotics, n (%)^a^	
Vancomycin	37 (66.1)
Piperacillin–tazobactam	35 (62.5)
Ceftaroline	17 (30.4)
Meropenem	15 (26.8)
Metronidazole	9 (16.1)
Levofloxacin	9 (16.1)
Ceftriaxone	8 (14.3)
Azithromycin	7 (12.5)
Ampicillin–sulbactam	7 (12.5)
Tigecycline	7 (12.5)
Clindamycin	4 (7.1)
Doxycycline	3 (5.4)
Linezolid	3 (5.4)
Aztreonam	2 (3.6)
Daptomycin	1 (1.8)
Penicillin	1 (1.8)
Polymixin B	1 (1.8)
Number of intrapleural administrations of alteplase/dornase alfa, n (%)	
One	5 (8.9)
Two	6 (10.7)
Three	40 (71.4)
Four	1 (1.8)
Five	1 (1.8)
Six	3 (5.4)
Patients who received the first dose of intrapleural treatment in under 24 h after chest tube insertion, n (%)	37 (66.1)
Median days from chest tube insertion to first dose of intrapleural treatment (IQR)	1 (0–2)

^a^ All patients received more than one antibiotic during treatment

#### Outcomes

Outcome data is presented in Table 3. Fifty-two out of fifty-six (92.9%) of the patients experienced treatment success. The median number of days from chest tube insertion to first dose of intrapleural treatment was 1 day (IQR, 0–2) with 66.1% receiving the first dose of intrapleural tPA/DNase within 24 h. The median chest tube outputs at 24 h and 72 h after intrapleural therapy were 447.5 mL (IQR, 177.5–769.8) and 1470 mL (IQR, 717.5–1989.8), respectively. Median chest tube duration was 10 days (IQR, 7–14) and median hospital length of stay was 15 days (IQR, 11–28).

**Table 3:** Clinical outcomes and adverse events

Treatment outcomes	Data
Treatment success, n (%)	52 (92.9)
Surgical intervention for treatment failure (VATS with decortication), n (%)	2 (3.6)
30 day mortality due to pleural infection, n (%)	2 (3.6)
30 day readmission rate, n (%)	0 (0)
Median hospital stay (IQR)	15 (11–28)
Median cumulative volume of pleural drainage 24 h after first dose of intrapleural therapy (IQR), mL	447.5 (177.5–769.75)
Median cumulative volume of pleural drainage 48 h after first dose of intrapleural therapy (IQR), mL	995 (520–1445.5)
Median cumulative volume of pleural drainage 72 h after first dose of intrapleural therapy (IQR), mL	1470 (717.5–1989.75)
Median days of chest tube in pleural cavity (IQR)	10 (7–14)

Bleeding outcomes	
Pleural bleeding	9 (16.1)
Pleural bleeding requiring ≥ 2U pRBC transfusion	5 (8.9)
Gastrointestinal bleeding	0 (0)
Intracranial bleeding	0 (0)
Other bleeding	0 (0)
Mean hemoglobin loss (SD), g/dL	3.5 (1.1)
Median units of pRBC transfusion in patients transfused (IQR)	2 (2–5)
Surgical intervention for pleural bleeding, n (%)	3 (5.4)
30 day mortality due to pleural bleeding, n (%)	0 (0)

Four patients (7.1%) experienced treatment failure, 2 of which received surgical intervention. These two patients underwent video assisted thoracoscopic surgery (VATS) with decortication and both were successfully discharged and survived beyond 30 days. The other two patients with treatment failure had refractory septic shock and were not considered operative candidates.

#### Adverse events

Nine patients (16.1%) experienced pleural bleeding as defined in “Methods” section. Of these, only five (8.9% of all patients) developed pleural bleeding significant enough to require transfusion of at least 2 units of pRBC. Three of these five patients (5.4% of all patients) required surgical intervention with VATS for evacuation of hematoma. No patients experienced major bleeding at any other predefined sites.

All nine patients who experienced pleural bleeding received three or more doses of tPA/DNase. Mean hemoglobin loss was 3.5 g/dL (± 1.1) and the median units of pRBC transfusions was 2 units (IQR 2–5). There was no mortality related to pleural bleeding at 30 days. Of the five patients requiring ≥ 2 units of pRBC, one was on full dose anticoagulation (rivaroxaban), which was held for 2 days prior to chest tube insertion but was resumed during the time this patient received fibrinolytic therapy. Three patients were on aspirin alone. In comparison, of the 47 patients who had no evidence of pleural bleeding while receiving tPA/DNase, 21 patients (44.7%) were on some form of antiplatelet agent or an anticoagulant (12 patients on aspirin alone, 4 patients on aspirin and clopidogrel, 1 patient on rivaroxaban, 1 patient on apixaban, 1 patient on enoxaparin, and 1 patient on warfarin).

The 30-day mortality rate due to pleural infection was 3.6% (2 out of 56 patients). These two patients both received three doses of tPA/DNase and passed away at days 14 and 27. Both were in the intensive care unit setting with septic shock due to empyema and acute respiratory failure requiring mechanical ventilation.

### Discussion

Empyema and CPE are associated with significant morbidity and mortality [1, 2, 8]. Intrapleural administration of tPA (with or without DNase) at 10 mg has been reported to lead to pleural bleeding at rates between 1.8 and 12.0% [5, 9–13]. Based on one pharmacologic study, the bleeding risk of intrapleural tPA appears to be dose dependent [13].

The MIST-2 trial [4], which utilized 10 mg of alteplase and 5 mg of dornase alfa twice daily, demonstrated a 3.8% rate of pleural bleeding. However, a retrospective study using the same dosing regimen [13], led to a pleural bleeding rate of 5.4%. There is conflicting data as to whether reducing the dose of tPA will lead to a reduction in pleural bleeding [14, 15].

Conversely, a study utilizing extended tPA/DNase dosing (mean 9.8 doses) in patients who were not surgical candidates, demonstrated a similar efficacy rate as six doses but a higher pleural bleeding rate at 10% [16]. Two studies investigated the effect of concurrent administration of tPA and DNase. One study showed comparable treatment success rates but an increased rate in pleural bleeding as compared to MIST-2 [13]. The other showed treatment success comparable to MIST-2 trial but with no pleural bleeding [15].

Our data shows that a concurrent once daily dosing strategy was associated with a similar rate of treatment success as previous studies that utilized a twice daily, sequential dosing strategy. Rates of treatment failure requiring VATS decortication in our cohort (5.4%) were comparable to the rates found in previous studies of 4.0 to 9.6% [4, 5, 13]. Given apparent similarities in the clinical and biochemical characteristics of our patient cohort in comparison to these other studies, it is reasonable to conclude that a once daily concurrent dosing regimen is as effective as twice daily concurrent dosing.

Although our treatment success rate was comparable to that of previously reported studies, our patients experienced a longer duration of chest tube therapy and length of hospital stay compared to prior studies. This raises the possibility that the reduced dosing or the concurrent administration of medications leads to a longer duration of drainage time before completion of therapy. Alternatively, this could reflect variations in local treatment practices or the absence of rigid protocols of a clinical trial setting that may have led to earlier removal of chest tubes.

Despite using a lower total dose of alteplase, our study revealed an overall pleural bleeding rate of 16.1%. The rate of pleural bleeding requiring at least 2 units of pRBC was 8.9% (5 patients). Three patients (5.4%) required VATS for hematoma evacuation. This stands in contrast to previous studies which showed overall rates of pleural bleeding ranging from 0 to 5.4% and no reports of pleural bleeding requiring VATS for hematoma evacuation [4, 5, 9, 13, 15, 16]. The use of antiplatelet or anticoagulant medications may have led to the increased bleeding rate. Further, we speculate there may be a synergistic effect of concurrent administration of tPA/DNase leading to the increased pleural bleeding rate.

### Conclusion

In this single center, retrospective study of patients presenting with complicated parapneumonic effusion or empyema, the use of once daily concurrently administered tPA/DNAse led to comparable rates of the primary efficacy outcome of treatment success as prior studies utilizing twice daily concurrent or sequential dosing.

## Limitations

- This retrospective design and relatively small sample size limits definitive conclusions.
- A component of our definition of clinical success included physician assessment, which is subjective in nature.
- Assessment of radiographic improvement was based on radiologist interpretation and not by objective quantitative means, such as CT volumetry.

## Data Availability

The datasets used and analyzed during the current study are available from the corresponding author on reasonable request.

## Abbreviations

CPE: Complicated parapneumonic effusion
CRP: C-reactive protein
CT: Computed tomography
DNase: Dornase alfa
ESR: Erythrocyte Sedimentation Rate
IQR: Inter-quartile range
LDH: Lactate dehydrogenase
pRBC: Packed red blood cell
tPA: Tissue plasminogen activator
SD: Standard deviation
VATS: Video assisted thoracoscopic surgery
